# Cardiology referral during the COVID-19 pandemic

**DOI:** 10.6061/clinics/2021/e3538

**Published:** 2021-11-23

**Authors:** Nathalia Conci Santorio, Francisco Akira Malta Cardozo, Rodrigo Freddi Miada, Fabio Grunspun Pitta, Caio de Assis Moura Tavares, Fabio Cetinic Habrum, Henrique Trombini Pinesi, Iuri Resedá Magalhães, Maria Clara Saad Menezes, Bruno Caramelli, Daniela Calderaro

**Affiliations:** Instituto do Coracao (InCor), Hospital das Clinicas HCFMUSP, Faculdade de Medicina, Universidade de Sao Paulo, Sao Paulo, SP, BR.

**Keywords:** Cardiology Referral, COVID-19, Myocardial Injury, Referral, Cardiology Training

## Abstract

**OBJECTIVES:**

This study presents the cardiology referral model adopted at the University of São Paulo-Hospital das Clínicas complex during the initial period of the coronavirus disease (COVID-19) pandemic, main reasons for requesting a cardiologic evaluation, and clinical profile of and prognostic predictors in patients with COVID-19.

**METHODS:**

In this observational study, data of all cardiology referral requests between March 30, 2020 and July 6, 2020 were collected prospectively. A descriptive analysis of the reasons for cardiologic evaluation requests and the most common cardiologic diagnoses was performed. A multivariable model was used to identify independent predictors of in-hospital mortality among patients with COVID-19.

**RESULTS:**

Cardiologic evaluation was requested for 206 patients admitted to the ICHC-COVID. A diagnosis of COVID-19 was confirmed for 180 patients. Cardiologic complications occurred in 77.7% of the patients. Among these, decompensated heart failure was the most common complication (38.8%), followed by myocardial injury (35%), and arrhythmias, especially high ventricular response atrial fibrillation (17.7%). Advanced age, greater need of ventilatory support on admission, and pre-existing heart failure were independently associated with in-hospital mortality.

**CONCLUSIONS:**

A hybrid model combining in-person referral with remote discussion and teaching is a viable alternative to overcome COVID-19 limitations. Cardiologic evaluation remains important during the pandemic, as patients with COVID-19 frequently develop cardiovascular complications or decompensation of the underlying heart disease.

## INTRODUCTION

Cardiology referral is an essential occupation area and a good opportunity for the technical training of cardiologists. It consists of support to other medical specialties in managing cardiovascular problems associated with primarily non-cardiac diseases such as infectious, respiratory, or systemic diseases and in preparation for non-cardiac surgeries (1). Cardiology referral is associated with improved clinical outcomes in addition to the opportunity to diagnose other diseases previously unknown to patients or the requesting team (2).

In the initial period of the coronavirus disease (COVID-19) pandemic, the University of São Paulo School of Medicine’s Hospital das Clínicas complex (HC-FMUSP) was restructured to attend only the suspected or confirmed COVID-19 cases in its 900-bed Central Institute to optimize care, reduce intra-hospital contamination, and meet the growing demand of the Unified Health System for isolation beds.

During this period, cardiology referral also underwent changes, since there was a greater demand associated with the cardiovascular complications of COVID-19. A significant increase in the number of patients requiring intensive care, new challenges imposed by the need to restrict the movement of personnel, and the rational use of personal protective equipment were important challenges during this phase. In this scenario of relocation for assistance, it was also necessary to continue supervision and guidance of resident physicians in cardiology.

This study aimed to report the referral model adopted in our service and the main reasons for requesting a cardiologic referral for hospitalized COVID-19 patients. We also characterized the clinical profiles and prognostic predictors of the COVID-19 patients hospitalized at the HC-FMUSP for whom cardiology referrals were requested.

## METHODS

The main objective of the present study is to describe how cardiology consultation was performed in a quaternary university hospital in terms of patient care and medical training during the COVID-19 pandemic. All referral requests between March 30, 2020 and July 6, 2020 were eligible for inclusion in our study and encompassed suspected and confirmed COVID-19 patients. In this observational cohort, data were obtained and managed prospectively using the REDCap tool. Initial bedside assessments were performed by cardiology residents of the Heart Institute who were organized in pairs on a monthly rotation basis and supervised in person by attending physicians without risk factors for developing the most severe forms of the disease. This team was exclusively dedicated to care for COVID-19 patients.

A daily videoconference was held among regional physicians dedicated to COVID-19 care and other physicians allocated to areas without infected patients through the InCor platform for telemedicine to ensure confidentiality of information. Clinical, laboratory, and imaging data of the most severe cases were reviewed during the videoconference and the best treatment plan was selected *via* a joint decision of the group.

The reasons for cardiologic evaluation request were defined according to the referral by the assisting team and not the final cardiologic diagnosis.

The diagnosis of COVID-19 was confirmed using laboratory criteria (positive reverse transcription-polymerase chain reaction [RT-PCR] or serology test result) or clinical criteria (clinical presentation and chest computed tomography findings compatible with the diagnosis of COVID-19). Patients without a confirmed diagnosis of COVID-19 were excluded from the analysis of clinical characteristics, cardiovascular manifestations, complementary examinations, and main clinical outcomes.

Troponin elevation was defined as the level of high-sensitive cardiac troponin T above the 99th percentile of normality, which characterizes myocardial injury. Troponin elevation was considered acute if there was an increase or decrease in its levels and chronic when the elevated levels plateaued. Diagnosis of infarction was established according to the Fourth Universal Definition of Acute Myocardial Infarction (3).

### Statistical analysis

Descriptive statistics included the reasons for the cardiologic evaluation request. For exploratory data analysis, we presented the primary clinical, demographic, and laboratory characteristics and clinical outcomes during hospitalization according to in-hospital mortality outcomes. The variables included sex, age, previous comorbidities, use of medications, laboratory tests at admission (troponin, N-terminal pro b-type natriuretic peptide, D-dimer, C-reactive protein [CRP], ferritin, and lactate dehydrogenase), and main electrocardiographic presentations. The clinical outcomes considered for analysis included the need for hemodialysis, need for mechanical ventilation, use of vasoactive drugs, sepsis, acute heart failure, arrhythmias, troponin elevation, and acute myocardial infarction (AMI).

Continuous variables with normal distribution are presented as means and standard deviations, while variables with skewed distribution are presented as medians and interquartile ranges. In the univariate analysis, the unpaired t-test was used to compare the means of continuous variables between patients who died during hospitalization and those who were discharged. The Mann-Whitney test was used for variables with a skewed distribution. The chi-square test was used to compare frequencies of categorical variables with respect to death during hospitalization. Pairwise deletion was applied to remove cases with missing data. A multivariable logistic regression model was used to identify independent predictors of mortality. It consisted of variables that could be measured at hospital admission. Variables identified as significant in the univariate analyses (*p*<0.05) were included in this model. All tests were performed using PASW Statistics for Windows, version 18.0 (SPSS Inc, Chicago, IL, USA), with a significance level of 5%, two-tailed probability, and a confidence interval of 95%.

## RESULTS

**Figure 1:**
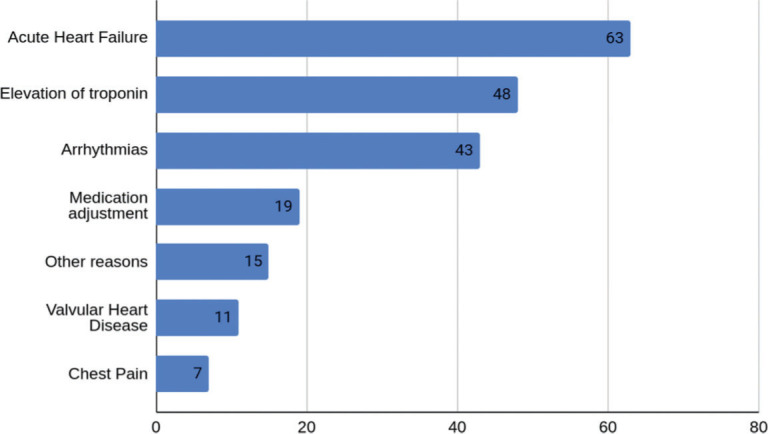
Reasons for requesting a cardiology referral. Other reasons: syncope, pericardial effusion, congenital heart disease, hypertension, and endocarditis. Data labels display the absolute number of patients.

During the study period, cardiologic referrals were requested for 206 patients admitted to the ICHC-COVID. The most frequent reasons for requesting cardiologic evaluation (Figure 1) were acute heart failure in 63 patients (30.6%); elevation of troponin in 48 patients (23.3%); arrhythmias in 43 patients (20.9%); need for adjustment in cardiovascular medication in 19 patients (9.2%); valvular heart disease evaluation in 11 patients (5.3%); chest pain without troponin elevation in 7 patients (3.3%); and syncope, pericardial effusion, congenital heart disease, hypertension, or endocarditis in 15 patients (7.28%).

The diagnosis of COVID-19 was confirmed in 87.4% of the patients (using laboratory criteria for 76.7% and clinical/radiological criteria for 10.7% of the patients). Overall, 12.6% of the patients tested negative for COVID-19 after clinical and laboratory re-evaluation, mainly with an alternative diagnosis of acute heart failure. Analysis of the prognosis and clinical and laboratory characteristics was performed for 180 patients with confirmed COVID-19.

The mean age was 62 years (60.8% men). The most frequent comorbidities were hypertension (77.2%), diabetes (42.2%), heart failure (39.4%), and coronary artery disease (32.8%). Table 1 shows the clinical and demographic characteristics of the population with respect to death during hospitalization. The mean duration between the onset of symptoms and cardiologic evaluation was 12 days. The most common symptoms were dyspnea (77.2%), cough (63.3%), and fever (51.7%), and chest pain (8.3%). At admission, 18.3% of the patients were intubated, 2.2% were receiving high-flow oxygen therapy, 7.8% were on non-invasive mechanical ventilation, and only 19.4% could breathe normally. Based on tomographic findings assessed by a radiologist, 24.4% of the patients exhibited involvement of more than 50% of the lung parenchyma and peripheral ground-glass opacities constituted the most frequent pattern (89.4%).

The most commonly used medications during hospitalization were antibiotics (85.2%), oseltamivir (42.9%), corticosteroids (16.3%), and hydroxychloroquine (4.9%). For anticoagulation, 56.3% of the patients received low molecular weight or unfractionated heparin in a prophylactic dose, while 32.5% received full doses. Intermediate dose regimens of anticoagulants were used in 4.9% of the cases.

Missing data at admission included those of the following laboratory variables: troponin (16.6%), brain natriuretic peptide (48.3%), D-dimer (22.2%), CRP (15%), hemoglobin (3.3%), lymphocytes (9.4%), and platelets (5.5%).

**Figure 2:**
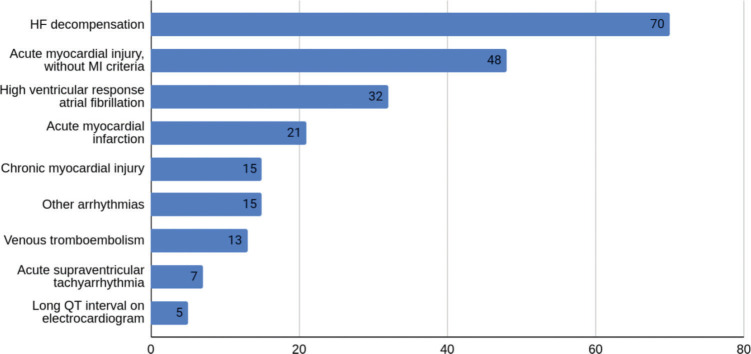
Cardiovascular manifestations in patients diagnosed with COVID-19. HF: heart failure, MI: myocardial infarction. Data labels display absolute number of patients.

The cardiology referrals revealed cardiologic complications in 77.7% of the patients diagnosed with COVID-19. The most frequent complications were decompensated heart failure, arrhythmias, and acute myocardial injury (Figure 2). There were no cases of acute myocarditis because of severe acute respiratory syndrome coronavirus 2 (SARS-CoV-2), and only one case of acute pericarditis was recorded.

The most frequent comorbidities noted among patients with COVID-19 and cardiologic manifestations were systemic arterial hypertension (76.2%), previous heart failure (43.4%), diabetes mellitus (43.4%), stable coronary artery disease (30.1%), and obesity (20.3%). Only 6.3% of the patients had no known comorbidities.

Among the 70 patients with acute heart failure, 15.7% had been diagnosed with heart failure with preserved ejection fraction and 48.6% had been diagnosed with heart failure with reduced ejection fraction.

Altogether, 84 cases of troponin elevation were noted. Among these, 25% had AMI, 57.1% had non-ischemic acute myocardial injury, and 17.9% had chronic myocardial injury. Among patients with acute myocardial injury (ischemic or non-ischemic), 40.6% had been previously diagnosed with chronic coronary artery disease.

**Figure 3:**
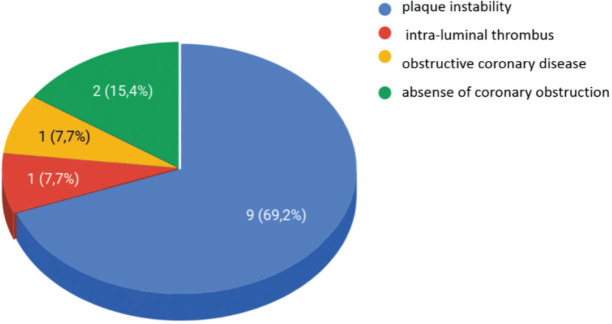
Obstructive patterns found in patients with coronavirus disease and acute myocardial infarction submitted to invasive stratification.

All patients diagnosed with AMI underwent transthoracic echocardiography during hospitalization. Among these, 47.6% had ventricular ejection fraction below 50% with segmental dysfunction. Invasive stratification was performed using cineangiocoronariography for 13 patients, revealing plaque instability and/or intraluminal thrombus in 77% of the cases. The angiographic characteristics are shown in Figure 3. ST-segment elevation was recorded in five patients (23.8%). ST-segment elevation myocardial infarction occurred in the anterior wall in all cases, and angioplasty of the anterior descending artery was successfully performed in four patients. According to the shared decision of the attending teams, clinical instability that precluded transport to the catheterization department was the main reason for not performing invasive stratification in all cases of AMI.

The triple-vessel atherosclerotic involvement pattern was found in 46.1% of the patients who underwent coronary angiography. The single-arterial pattern was observed in 23% of the patients, while 15.4% exhibited the two-arterial pattern. Angioplasty was performed in nine patients (42.8%), while others remained under medical treatment.

Non-cardiac complications were frequent, highlighting the need for mechanical ventilation (39.9%), sepsis (33.7%), hemodialysis (16.3%), and use of vasoactive drugs (42.7%).

On worsening of the clinical condition, 65% of the cases required intensive care unit admissionand 20.6% of the evaluated patients died. Table 2 shows the main clinical outcomes (cardiac and non-cardiac complications) and associated mortality.

For the multivariable analysis, we considered only parameters that could be measured at hospital admission. The following variables were included in the logistic regression model: age, pre-existing heart failure, chronic need for dialysis, degree of need for ventilatory support, lymphocytes, platelets, and initial troponin level (Table 1). The variables independently associated with in-hospital mortality were advanced age (relative risk [RR]: 1.06, 95% CI: 1.01-1.11, *p*=0.021), greater need for ventilatory support on admission (RR: 1.83, 95% CI: 1.24-2.72; *p*<0.003), and pre-existing heart failure (RR: 3.38, 95% CI: 1.02-11.17, *p*=0.046).

## DISCUSSION

In the present study, we demonstrated our experience in maintaining specialized cardiology care for patients with COVID-19, ensuring supervision and teaching of resident physicians, and minimizing the risk of contamination between teams and patients. The need for isolation of COVID-19 patients involves restricting movement of patients and personnel and rationally using protective equipment. These factors hinder the usual format of in-person bedside visits by residents, preceptors, and attending physicians. In this scenario, auxiliary use of telemedicine has played an essential role in optimizing care and safeguarding health teams (4). Among several specialties, experiences associated with dermatology (5), nephrology (6), and palliative care (7) have been reported. Although digital tools are better established in the outpatient environment, they can also be adopted in the in-hospital environment, reducing the need for in-person assessments by all healthcare team members. However, owing to the severity and complexity of cardiologic manifestations in patients with COVID-19, additional information from physical examinations and bedside decision-making is required every day. Thus, an in-person evaluation model involving case discussion and remote teaching was adopted to provide a better risk *versus* benefit ratio for patients and healthcare professionals.

Our experience with referral for patients with COVID-19 enabled residents’ practical and theoretical training in pathologies highly relevant to clinical practice such as acute coronary syndrome, arrhythmias, and heart failure, demonstrating that training can be achieved safely and effectively with proper adaptations (8,9).

In daily practice of cardiology consultation during a pandemic, challenges begin with the appropriate diagnosis of COVID-19. RT-PCR for the diagnosis of COVID-19 has exhibited an approximate sensitivity of 86% and a specificity of 96% (10). In patients with previous heart disease, tomographic findings of pulmonary congestion may be difficult to differentiate from viral involvement and symptoms such as cough and dyspnea, which can be easily attributed to heart failure. In this scenario, cardiology referral excluded the diagnosis of COVID-19 in 12.6% of the cases. The exploratory statistical analysis of the study population characteristics identified pre-existing heart failure as a predictor of in-hospital mortality (a three times greater risk of death). This result was consistent with worldwide epidemiological findings, which suggested that the presence of cardiovascular comorbidities was associated with a worse prognosis (11). The prevalence of previously known cardiac comorbidities was high (heart failure: 39.4%, coronary disease: 32.8%, hypertension: 77%, and atrial fibrillation: 18.9%), which can be explained by the fact that we evaluated only cases screened by other medical specialties. However, such comorbidities were not related to mortality. We believe that patients referred for cardiological evaluation might have exhibited an attenuated impact of other cardiovascular comorbidities on prognosis.

Acute myocardial injury and differential diagnosis of acute coronary syndrome were among the most frequent reasons for referral requests. Acute myocardial injury was observed in 38.3% of the confirmed cases of COVID-19, and only a quarter of patients with troponin elevation were diagnosed with AMI. Reportedly, the general incidence of acute myocardial injury was approximately 17% among hospitalized patients and up to 59% of these patients died (12). Acute troponin elevation was observed in 89.3% of the patients who died subsequently. Troponin elevation has prognostic implications. It is associated with a significant increase in mortality (51.2% *versus* 4.5%) as well as a higher incidence of severe acute respiratory syndrome (58.5% *versus* 14.7%), mechanical ventilation (22% *versus* 4.2%), and acute kidney injury (8.5% *versus* 0.3%) when compared with COVID-19 patients with normal troponin levels (13). The impact of elevated troponin levels on mortality is even worse among COVID-19 patients with a previous history of cardiovascular disease (14). The high prevalence of structural heart diseases in the population screened for cardiology referral may explain the higher prevalence of myocardial injury in our sample.

In the present study, AMI was confirmed in 21 patients. Altogether, 62% of patients underwent invasive stratification and only two patients did not exhibit obstructive lesions, confirming the validity of early specialized evaluation of critically ill patients with COVID-19. A Brazilian study evaluated 152 patients diagnosed with infarction and COVID-19 and demonstrated multivessel disease in 69% of the patients, with a mortality rate of 23.7%. ST-segment elevation was observed in 54.6% of the cases, and 18.4% of them had a previous diagnosis of coronary artery disease (15).

The greatest strength of the present study is utilization of an efficient model of cardiologic referral practice in a teaching hospital. This model involved referrals for complex cases of COVID-19, with due consideration to medical care for patients as well as medical training of the residents. The exploratory statistical analysis was limited to a small inpatient population diagnosed with COVID-19. Thus, its conclusions should not be extrapolated to the majority of the population infected with SARS-CoV-2, which remains outside the hospital environment. Moreover, this study was conducted before the availability of vaccines against SARS-CoV-2, which might modify the clinical parameters and outcomes of these patients.

## CONCLUSIONS

A hybrid model involving in-person referrals with remote discussion and teaching is a viable alternative for reducing the movement of patients and personnel. It helps maintain the wellbeing of patients and health teams with less burden on care providers and ensures practical and theoretical training of cardiology residents. Patients with COVID-19 frequently present with new cardiovascular involvement or decompensation of the underlying heart disease. Specialized cardiologic evaluation is vital to assist in the management of such cases.

## AUTHOR CONTRIBUTIONS

Calderaro D was responsible for the study design. Santorio NC, Cardozo FAM, Miada RF, Pitta FG, Tavares CAM, Habrum FC, Pinesi HT, Menezes MCS and Magalhães IR collected the data. Santorio NC, Cardozo FAM, and Miada RF were responsible for data retrieval and manuscript drafting. Calderaro D performed statistical analyses. Calderaro D and Caramelli B were responsible for reviewing the manuscript. All of the authors have approved the final version of the manuscript.

## Figures and Tables

**Table 1:** Clinical and demographic data and complementary examinations at admission.

	Total n=180 (100%)	Death on admission n=37 (20.5%)	Hospital discharge n=143 (79.5%)	*p*-value
Sex				
Male (%)	109 (60.5%)	22 (59.5%)	87 (60.8%)	0.99
Age, mean (SD)	62.1 (14.6)	68.6 (10.8)	60.4 (14.9)	<0.001
Prior comorbidities, n (%)				
Hypertension	139 (77.2%)	29 (78.4%)	110 (76.9%)	0.851
Heart failure	71 (39.4%)	21 (56.7%)	50 (34.9%)	0.023
Current or previous smoking	61 (34.1%)	13 (36.1%)	48 (33.6%)	0.541
Coronary artery disease	59 (32.8%)	16 (43.2%)	43 (30.1%)	0.128
Non-insulin-dependent diabetes mellitus	54 (30%)	14 (37.8%)	40 (28%)	0.243
Obesity	34 (18.9%)	5 (13.5%)	29 (20.3%)	0.349
Atrial fibrillation	34 (18.9%)	10 (27%)	24 (16.8%)	0.156
Non-dialysis chronic kidney disease	29 (16.1%)	4 (10.8%)	25 (17.5%)	0.325
Insulin-dependent diabetes mellitus	22 (12.2%)	5 (13.5%)	17 (11.9%)	0.788
Moderate/important valve disease	17 (9.4%)	4 (10.8%)	13 (9.1%)	0.750
Chronic obstructive pulmonary disease	13 (7.3%)	4 (11.1%)	9 (6.3%)	0.344
Dialysis for chronic kidney disease	10 (5.6%)	5 (13.5%)	5 (3.5%)	0.018
Prior use of medications, n (%)				
ACEi/ARA	101 (57.4%)	23 (62.1%)	78 (56.1%)	0.671
Beta-blockers	76 (43.2%)	20 (54.1%)	56 (40.3%)	0.102
Statins	72 (40.9%)	19 (51.4%)	53 (38.1%)	0.114
Aspirin	49 (27.8%)	10 (27%)	39 (28.1%)	0.976
Oral hypoglycemic agents	41 (23.3%)	10 (27%)	31 (22.3%)	0.489
Warfarin	19 (10.8%)	5 (13.5%)	14 (10.1%)	0.511
P2Y12 inhibitors	14 (8%)	5 (13.5%)	9 (6.5%)	0.144
Direct oral anticoagulants	4 (2.3%)	2 (5.4%)	2 (1.4%)	0.141
Laboratory examinations on admission				
Troponin, median (IQR)	0.048 (0.004-10.225)	0.078 (0.011-4.91)	0.035 (0.004-10.225)	0.002
NT-proBNP, median (IQR)	3343.5 (5-125965)	11529.5 (8372-35516)	2743 (5-125965)	0.05
D-dimer, median (IQR)	1626.5 (190-114452)	1526 (190-95284)	1743 (225-114452)	0.58
CRP, median (IQR)	98.5 (1.1-458.3)	128.25 (1.1-458.3)	96 (1.92-422)	0.80
Lymphocytes, mean (SD)	988 (585)	771.42 (583)	1044 (575)	0.036
Platelets, mean (SD)	223708 (90806)	180595 (69890)	235021 (92460)	0.001
Hemoglobin, mean (SD)	12.03 (2.29)	11.66 (2.64)	12.13 (2.18)	0.276
Electrocardiography findings on admission (%)				
Atrial fibrillation	36 (20.1%)	12 (32.4%)	24 (16.9%)	0.04
Normal	14 (7.8%)	0 (0%)	14 (9.9%)	0.08
T wave inversion, ST depression, or pathological Q wave	11 (6.1%)	6 (16.2%)	5 (3.5%)	0.22
ST-segment elevation	5 (2.8%)	1 (2.7%)	4 (2.8%)	0.97
Long QT interval	5 (2.8%)	0 (0%)	5 (3.5%)	0.58
O_2_ requirement at admission				
Nasal cannula	94 (52.2%)	13 (35.1%)	81 (56.6%)	
Ambient air	35 (19.4%)	3 (8.1%)	32 (22.4%)	<0.001
Mechanical ventilation	33 (18.3%)	12 (32.4%)	21 (14.7%)	
Non-invasive ventilation	14 (7.8%)	8 (21.6%)	6 (4.2%)	
High-flow nasal cannula	4 (2.2%)	1 (2.7%)	3 (2.1%)	

SD: standard deviation, ACEi: angiotensin-converting enzyme inhibitors, ARA: angiotensin receptor antagonists, IQR: interquartile range, NT-proBNP: N-terminal pro b-type natriuretic peptide, CRP: C-reactive protein.

**Table 2:** Main clinical outcomes and associated mortality.

Clinical outcome n (%)	Mortality of patients who presented with the outcome (%)	Mortality of patients who did not present with the outcome (%)	RR (95% CI)	*p*-value
Non-cardiac complications				
Hemodialysis 29 (16.1%)	55.2%	13.9%	7.62 (3.21-18.09)	<0.001
Sepsis 60 (33.5%)	50%	5.8%	16.14 (6.46- 40.31)	<0.001
Need for mechanical ventilation 71 (39.4%)	45.1%	4.6%	16.74 (6.08-46.06)	<0.001
Use of vasoactive drugs 77 (42.7%)	42.9%	3.9%	13.22 (5.34- 32.74)	<0.001
ICU admission 117 (65%)	29.1%	4.8%	8.19 (2.40- 27.92)	<0.001
Cardiac complications				
AMI 21 (11.7%)	38.1%	18.2%	2.33 (0.86-6.35)	0.090
Myocardial injury without AMI criteria 63 (35%)	36.2%	10.8%	4.50 (1.89-10.70)	<0.001
Acute HF 70 (38.8%)	24.3%	18.2%	1.44 (0.70-3.00)	0.323
HRAFib 32 (17.7%)	18.8%	20.9%	0.87 (0.33-2.30)	0.780

RR: relative risk, CI: confidence interval, ICU: intensive care unit, AMI: acute myocardial infarction, HF: heart failure, HRAFib: high-rate atrial fibrillation.
